# Supplementary material for: fluff: exploratory analysis and visualization of high-throughput sequencing data
Source: PeerJ. 2016 Jul 19;4:e2209. doi: 10.7717/peerj.2209 (PMC4957989; doi:10.7717/peerj.2209)
Supplement: Supplemental Information 1 [file peerj-04-2209-s002.pdf]

Supplemental Information

fluff: exploratory analysis and visualization of high-throughput sequencing data

Georgios Georgiou[1] and Simon J. van Heeringen[1,]*

[1]Radboud University, Department of Molecular Developmental Biology, Faculty of Science, Radboud Institute for Molecular Life Sciences, 6500HB Nijmegen, The Netherlands
*To whom correspondence should be addressed.

Contact: s.vanheeringen@science.ru.nl

Supplemental Figures

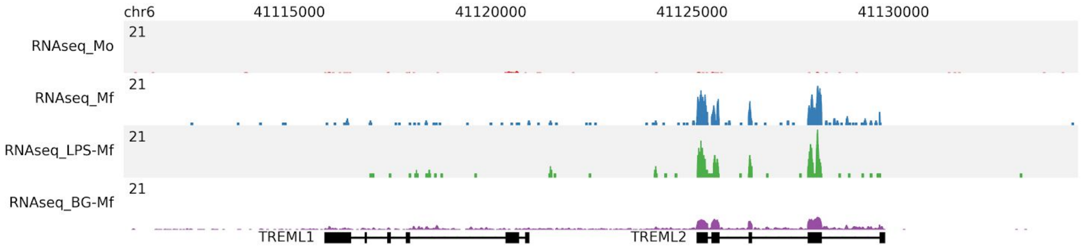

Supplemental Figure 1. Visualization of RNA-seq data using *fluff profile*. Shown are the RNA-seq profiles at the TREML1 and TREML2 gene loci of Monocytes (red), Macrophages (blue), Macrophages pre-incubated with LPS (green) and Macrophages pre-incubated with β-glucan (purple). Read depth (per million reads) is normalized to the total number of mapped reads per sample.

Supplemental Methods

Implementation

The fluff module and command-line tools are implemented in Python and make use of the following packages:

- colorbrewer (Brewer, 2016)
- HTSeq (Anders et al., 2015)
- pybedtools (Dale et al., 2011; Quinlan and Hall, 2010)
- pysam (Li et al. (2009); pysam - htslib interface for python)
- pycluster (de Hoon et al., 2004)

In addition fluff uses the numpy, scipy and matplotlib Python libraries.

The package can be installed using the Python package manager *pip* or the *conda* package manager from the Anaconda open source analytics platform (https://continuum.io). The source code is freely available at http://github.com/simonvh/fluff under a MIT license.

Data description

The H3K27ac ChIP-seq and RNA-seq data that were used for the monocyte-macrophage analysis (Fig. 1; Fig. S1) were downloaded from NCBI GEO (Edgar et al., 2002), accession GSE58310, and are described in Saeed et al. (2014). The specific samples are listed in Table 1. The ChIP-seq FASTQ files were mapped to the human genome (hg19) using bwa version 0.7.10 (Li and Durbin, 2009). The RNA-seq FASTQ files were mapped to the human genome (hg19) using gsnap version 2012-07-20 (Wu and Nacu 2010). Duplicate reads were marked using bamUtil 1.0.2 (http://genome.sph.umich.edu/wiki/BamUtil). The BAM files were filtered to remove all reads with mapping quality less than 15. The regions that were used as input for Figs. 1A and B are supplied in Supplementary Table 2.

The H3K27ac ChIP-seq and DNaseI data in human H1 cells (Fig. 2) were downloaded from NCBI GEO (Edgar et al., 2002), series accessions GSE18927 and GSE16256, and are described in Xie et al. (2013).. The FASTQ files were mapped to the human genome (hg19) using bwa version 0.7.12-r1039 (Li and Durbin, 2009). Duplicate reads were marked using bamUtil 1.0.2 (http://genome.sph.umich.edu/wiki/BamUtil). All BAM files from replicate experiments were merged. Peaks were called on the DNaseI BAM files using MACS2 2.1.0.20140616 (Zhang et al. (2008); https://github.com/taoliu/MACS/) with default settings. All DNaseI peaks of different experiments were merged and centered on the highest summit as determined by MACS2. The peaks and reads corresponding to chromosome 1 were filtered. This data set is available from figshare (van Heeringen, 2016; DOI:10.6084/m9.figshare.3113728.v1).

Command lines

To create the panels for Figure 1, Figure 2 and Supplementary Figure 1, fluff was run with the following settings:

Fig. 1A:
```
fluff heatmap -f dynamic_regions.bed -d Mo.bam Mf.bam LPS-Mf.bam
        BG-Mf.bam -C kmeans -k 5 -M pearson -g -e 12000 -T 20
        -o Saeed_dynamicRegions_Pearson_K5_e12000_g_T20
```

Fig. 1B:
```
fluff bandplot -f Saeed_dynamicRegions_Pearson_K5_e12000_g_clusters.bed
        -counts Saeed_dynamicRegions_Pearson_K5_e12000_g_readCounts.txt
        -s 1:4 -P 98.5 -T 20
        -o Saeed_dynamicRegions_Pearson_K5_e12000_g_T20_bandplot_T20
```

Fig. 1C:

```
fluff profile -i chr2:68495000-68551000 -d Mo.bam Mf.bam LPS-Mf.bam
       BG-Mf.bam -s 1:4 -T 10 -a hg19_geneAnnotation.bed
       -o CNRIP1_profile_chr2_68495000_68551000_T10
```

Fig. 2A:

```
fluff heatmap -f example_peaks.bed -d H1.bam mesenchymal.bam mesendoderm.bam
       neuronal_progenitor trophoblast.bam  -C k -k 7 -o H3K27ac_kmeans7 -P 5
```

Fig. 2B:

```
fluff heatmap -f example_peaks.bed -d H1.bam mesenchymal.bam mesendoderm.bam
       neuronal_progenitor trophoblast.bam -C k -k 7 -g -M p -o
       H3K27ac_kmeans7_dynamics
```

Fig. S1:

```
fluff profile -i chr6:41112015-41135714 -d RNAseq_Mo.bam RNAseq_Mf.bam
       RNAseq_LPS-Mf.bam RNAseq_BG-Mf.bam -a hg19_geneAnnotation.bed  -f 0 -s 1:4 -n
       -o RNAseq_TREML_chr6_41112015_41135714_f0_normalized
```

Supplemental Tables

Supplemental Table 2: List of H3K27ac ChIP-seq and RNA-seq samples used in this study

Experiment	Abbreviation	GEO accession
Monocytes	Mo	GSM1406295
Monocyte-derived Macrophage	Mf	GSM1406298
Monocyte-derived Macrophages pre-incubated with LPS	LPS-Mf	GSM1406301
Monocyte-derived Macrophages pre-incubated with β-glucan	BG-Mf	GSM1406304
H1 cell line	H1	GSM466732
H1 cell line	H1	GSM663427
H1 BMP4 derived mesendoderm cultured cells	mesendoderm	GSM753425
H1 BMP4 derived mesendoderm cultured cells	mesendoderm	GSM753426
H1 BMP4 derived mesendoderm cultured cells	mesendoderm	GSM864035

H1 BMP4 derived mesendoderm cultured cells	mesendoderm	GSM864799
H1 BMP4 derived trophoblast cultured cells	trophoblast	GSM753427
H1 BMP4 derived trophoblast cultured cells	trophoblast	GSM753428
H1 BMP4 derived trophoblast cultured cells	trophoblast	GSM864800
H1 derived neuronal progenitor cultured cells	neuronal progenitor	GSM753429
H1 derived neuronal progenitor cultured cells	neuronal progenitor	GSM767343
H1 derived neuronal progenitor cultured cells	neuronal progenitor	GSM818031
H1 derived neuronal progenitor cultured cells	neuronal progenitor	GSM896162
H1 derived neuronal progenitor cultured cells	neuronal progenitor	GSM956008
H1 derived mesenchymal stem cells	mesenchymal	GSM767341
H1 derived mesenchymal stem cells	mesenchymal	GSM767342
Monocytes RNA-seq	RNAseq_Mo	GSM1406318
Monocyte-derived Macrophage RNA-seq	RNAseq_Mf	GSM1406319
Monocyte-derived Macrophages pre-incubated with LPS RNA-seq	RNAseq_LPS-Mf	GSM1406320
Monocyte-derived Macrophages pre-incubated with β-glucan RNA-seq	RNAseq_BG-Mf	GSM1406321

Supplemental Table 3: List of DNaseI samples used in this study

Experiment	Abbreviation	GEO accession
H1 cell line	H1	GSM878616
H1 cell line	H1	GSM878621
H1 BMP4 derived mesendoderm cultured cells	mesendoderm	GSM817198
H1 BMP4 derived mesendoderm cultured cells	mesendoderm	GSM817215
H1 BMP4 derived trophoblast cultured cells	trophoblast	GSM878614
H1 BMP4 derived trophoblast cultured cells	trophoblast	GSM878628
H1 derived neuronal progenitor cultured cells	neuronal progenitor	GSM878615
H1 derived neuronal progenitor cultured cells	neuronal progenitor	GSM906379
H1 derived mesenchymal stem cells	mesenchymal	GSM906380
H1 derived mesenchymal stem cells	mesenchymal	GSM906381

References

Anders, S., Pyl, P. T., and Huber, W. (2015). HTSeq–a python framework to work with high-throughput sequencing data. Bioinformatics, 31(2):166–169.

BamUtil - Genome Analysis Wiki, http://genome.sph.umich.edu/wiki/BamUtil accessed on 2016-03-21

Brewer, C. (2016). ColorBrewer: Color advice for maps. http://www.colorbrewer2.org, accessed:2016-3-15.

Dale, R. K., Pedersen, B. S., and Quinlan, A. R. (2011). Pybedtools: a flexible python library for manipulating genomic datasets and annotations. Bioinformatics, 27(24):3423–3424.

de Hoon, M. J. L., Imoto, S., Nolan, J., and Miyano, S. (2004). Open source clustering software. Bioinformatics, 20(9):1453–1454.

Edgar, R., Domrachev, M., and Lash, A. E. (2002). Gene expression omnibus: NCBI gene expression and hybridization array data repository. Nucleic Acids Res., 30(1):207–210.

Li, H. and Durbin, R. (2009). Fast and accurate short read alignment with Burrows-Wheeler transform. Bioinformatics, 25(14):1754–1760.

Li, H., Handsaker, B., Wysoker, A., Fennell, T., Ruan, J., Homer, N., Marth, G., Abecasis, G., Durbin, R., and 1000 Genome Project Data Processing Subgroup (2009). The sequence Alignment/Map format and SAMtools. Bioinformatics, 25(16):2078–2079.

Saeed, S., Quintin, J., Kerstens, H. H. D., Rao, N. A., Aghajanirefah, A., Matarese, F., Cheng, S.-C., Ratter, J., Berentsen, K., van der Ent, M. A., Sharifi, N., Janssen-Megens, E. M., Ter Huurne, M., Mandoli, A., van Schaik, T., Ng, A., Burden, F., Downes, K., Frontini, M., Kumar, V., Giamarellos-Bourboulis, E. J., Ouwehand, W. H., van der Meer, J. W. M., Joosten, L. A. B., Wijmenga, C., Martens, J. H. A., Xavier, R. J., Logie, C., Netea, M. G., and Stunnenberg, H. G. (2014). Epigenetic programming of monocyte-to-macrophage differentiation and trained innate immunity. Science, 345(6204):1251086.

Quinlan, A. R. and Hall, I. M. (2010). BEDTools: a flexible suite of utilities for comparing genomic features. Bioinformatics, 26(6):841–842.

van Heeringen, S. J. (2016). fluff example data. Figshare. DOI:10.6084/m9.figshare.3113728.v1

Wu TD., Nacu S. 2010. Fast and SNP-tolerant detection of complex variants and splicing in short reads. Bioinformatics 26:873–881.

Xie, W., Schultz, M. D., Lister, R., Hou, Z., Rajagopal, N., Ray, P., Whitaker, J. W., Tian, S., Hawkins, R. D., Leung, D., Yang, H., Wang, T., Lee, A. Y., Swanson, S. A., Zhang, J., Zhu, Y., Kim, A., Nery, J. R., Urich, M. A., Kuan, S., Yen, C.-A., Klugman, S., Yu, P., Suknuntha,

K., Propson, N. E., Chen, H., Edsall, L. E., Wagner, U., Li, Y., Ye, Z., Kulkarni, A., Xuan, Z., Chung, W.-Y., Chi, N. C., Antosiewicz-Bourget, J. E., Slukvin, I., Stewart, R., Zhang, M. Q., Wang, W., Thomson, J. A., Ecker, J. R., and Ren, B. (2013). Epigenomic analysis of multilineage differentiation of human embryonic stem cells. Cell, 153(5):1134–1148.

Zhang, Y., Yong, Z., Tao, L., Meyer, C. A., Jérôme, E., Johnson, D. S., Bernstein, B. E., Chad, N., Myers, R. M., Myles, B., Wei, L., and Shirley Liu, X. (2008). Model-based analysis of ChIP-Seq (MACS). Genome Biol., 9(9):R137.
